# KRAS^G^ ^12C^‐inhibitor‐based combination therapies for pancreatic cancer: insights from drug screening

**DOI:** 10.1002/1878-0261.13725

**Published:** 2024-09-10

**Authors:** Constanza Tapia Contreras, Jonas Dominik Falke, Dana‐Magdalena Seifert, Carolin Schneider, Lukas Krauß, Xin Fang, Denise Müller, Engin Demirdizen, Melanie Spitzner, Tiago De Oliveira, Christian Schneeweis, Jochen Gaedcke, Silke Kaulfuß, Kimia Mirzakhani, Bernd Wollnik, Karly Conrads, Tim Beißbarth, Gabriela Salinas, Jonas Hügel, Nils Beyer, Sophia Rheinländer, Ulrich Sax, Matthias Wirth, Lena‐Christin Conradi, Maximilian Reichert, Volker Ellenrieder, Philipp Ströbel, Michael Ghadimi, Marian Grade, Dieter Saur, Elisabeth Hessmann, Günter Schneider

**Affiliations:** ^1^ Department of General, Visceral and Pediatric Surgery University Medical Center Göttingen Germany; ^2^ Institute of Pathology University Medical Center Göttingen Germany; ^3^ Institute for Translational Cancer Research and Experimental Cancer Therapy Technical University Munich Germany; ^4^ Clinical Research Unit 5002, KFO5002 University Medical Center Göttingen Germany; ^5^ Institute of Human Genetics University Medical Center Göttingen Germany; ^6^ Cluster of Excellence “Multiscale Bioimaging: From Molecular Machines to Networks of Excitable Cells” (MBExC) University of Göttingen Germany; ^7^ Department of Medical Bioinformatics University Medical Center Göttingen Germany; ^8^ CCC‐N (Comprehensive Cancer Center Lower Saxony) Göttingen Germany; ^9^ Campus‐Institute Data Science (CIDAS) Göttingen Germany; ^10^ NGS Integrative Genomics Core Unit (NIG) University Medical Center Göttingen (UMG) Germany; ^11^ Department of Medical Informatics University Medical Center Göttingen Germany; ^12^ Department of Hematology, Oncology and Cancer Immunology Campus Benjamin Franklin, Charité – Universitätsmedizin Berlin, Corporate Member of Freie Universität Berlin and Humboldt‐Universität zu Berlin Germany; ^13^ Medical Clinic and Polyclinic II, Klinikum rechts der Isar Technical University Munich Germany; ^14^ Translational Pancreatic Research Cancer Center, Medical Clinic and Polyclinic II, Klinikum rechts der Isar Technical University Munich Germany; ^15^ Center for Protein Assemblies (CPA) Technical University of Munich Garching Germany; ^16^ Center for Organoid Systems and Tissue Engineering (COS) Technical University Munich Garching Germany; ^17^ German Cancer Consortium (DKTK), Partner Site Munich, a Partnership Between DKFZ and University Hospital Klinikum rechts der Isar Munich Germany; ^18^ Department of Gastroenterology, Gastrointestinal Oncology and Endocrinology University Medical Center Göttingen Germany

**Keywords:** KRAS^G12C^, pancreatic cancer, SHP2, SOS1

## Abstract

Pancreatic ductal adenocarcinoma (PDAC) has limited treatment options, emphasizing the urgent need for effective therapies. The predominant driver in PDAC is mutated KRAS proto‐oncogene, *KRA*, present in 90% of patients. The emergence of direct KRAS inhibitors presents a promising avenue for treatment, particularly those targeting the *KRAS* ^*G12C*^ mutated allele, which show encouraging results in clinical trials. However, the development of resistance necessitates exploring potent combination therapies. Our objective was to identify effective KRAS^G12C^‐inhibitor combination therapies through unbiased drug screening. Results revealed synergistic effects with son of sevenless homolog 1 (SOS1) inhibitors, tyrosine‐protein phosphatase non‐receptor type 11 (PTPN11)/Src homology region 2 domain‐containing phosphatase‐2 (SHP2) inhibitors, and broad‐spectrum multi‐kinase inhibitors. Validation in a novel and unique KRAS^G12C^‐mutated patient‐derived organoid model confirmed the described hits from the screening experiment. Our findings propose strategies to enhance KRAS^G12C^‐inhibitor efficacy, guiding clinical trial design and molecular tumor boards.

## Graphical abstract

Through unbiased drug screening experiments in the context of pancreatic cancer, we observed that KRAS^G12C^ inhibitors combine favorably with inhibitors of the tyrosine phosphatase SHP2, a broad‐acting kinase inhibitor, and inhibitors of the guanine nucleotide exchange factor SOS1. These combinations are effective in a novel KRAS^G12C^‐driven patient‐derived organoid model.

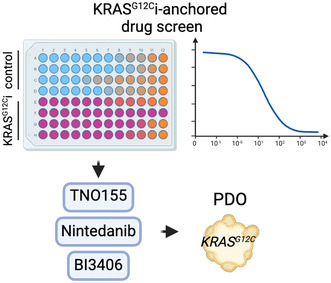

**Abbreviations**
AKT: protein kinase B
ARID1A: AT‐rich interaction domain 1A
ATR: ATR serine/threonine kinase
AUC: area under the dose–response curve
CDKN2A: cyclin dependent kinase inhibitor 2A
CRU5002: Clinical Research Unit 5002
DDR2: discoidin domain‐containing receptor 2
DNMT: DNA methyltransferase
EGFR: epidermal growth factor receptor
ERK: extracellular signal‐regulated kinase
FGFR1: fibroblast growth factor receptor 1
FGFR3: fibroblast growth factor receptor 3
GDP: guanosine diphosphate
GSEA: gene set enrichment analysis
GSVA: gene set variation analysis
GTP: guanosine‐5′‐triphosphate
IGFR1: insulin like growth factor 1 receptor
KRAS: KRAS proto‐oncogene, GTPase
MEK: mitogen‐activated protein kinase
MET: MET proto‐oncogene, receptor tyrosine kinase
NSCLC: non‐small cell lung cancer
PDAC: pancreatic ductal adenocarcinoma
PDCL: patient‐derived cell line
PDGFRA: platelet‐derived growth factor receptor alpha
PDGFRB: platelet‐derived growth factor receptor beta
PDO: patient‐derived organoid
PI3K: phosphatidylinositol‐3 kinase
PTPN11: tyrosine‐protein phosphatase non‐receptor type 11
RPK: reads per kilobase
SHP2: Src homology region 2 domain‐containing phosphatase‐2
SMAD4: SMAD family member 4
SOS1: son of sevenless homolog 1
ssGSEA: single sample gene set enrichment analysis
SWI/SNF: SWItch/sucrose non‐fermentable ATP‐dependent chromatin remodeling complex
TP53: tumor protein p53
TPM: transcripts per million
VEGFR1: vascular endothelial growth factor receptor1
VUS: variants of uncertain significance

## 1 Introduction

KRAS is a small GTPase that undergoes a cycle between an inactive GDP‐bound state and an active GTP‐bound state. In its active form, KRAS participates in both canonical MEK–ERK‐ and noncanonical PI3K‐AKT‐signaling pathways, which are controlling cell survival and proliferation [1]. Mutations in the KRAS oncogene, at codons 12, 13, 61, or 146, favor the active state of this GTPase. Notably, with 90%, KRAS mutations are highly prevalent in pancreatic ductal adenocarcinomas (PDACs), serving as a major driver of disease progression. Consequently, KRAS has emerged as a promising therapeutic target for PDAC. The most common mutations in KRAS are G12D (37%) and G12V (28%), whereas the G12C mutation, which is more frequently observed in non‐small cell lung cancer (NSCLC), occurs in only 1% of PDAC cases [1].

In recent years, there have been significant advancements in the discovery of novel and direct KRAS inhibitors [1, 2]. Currently, clinical development of KRAS^G12C^ inhibitors in PDAC is underway. In mono‐therapeutic regimens with sotorasib and adagrasib, response rates of 20–30% have been reported [3, 4]. Additionally, treatment with divarasib, a KRAS^G12C^ inhibitor with higher potency than sotorasib and adagrasib, resulted in partial responses in three out of seven PDAC patients treated [5]. However, the median progression‐free survival of 4.0–5.4 months highlights the rapid development of resistance [3, 4]. Therefore, it is crucial to increase the responding population and delay resistance development. Recognizing that combination therapies hold promise in addressing these challenges [6], we conducted a drug screening experiment to unbiasedly identify KRAS^G12C^ inhibitor combinations. Subsequently, we validated these combinations in a novel KRAS^G12C^‐mutated patient‐derived organoid (PDO) line. Here, we show that SOS1, SHP2, and multi‐kinase inhibitors are synergistic with KRAS^G12C^ inhibitors, strengthening clinical efforts to implement such therapeutic approaches for PDAC.

## 2 Materials and methods

### 2.1 Ethics statement

The primary human PDAC models were established and analyzed in accordance with the Declaration of Helsinki and were approved by the local ethical committee of the University Medical Center Göttingen (UMG) (vote 11/5/17). Written informed consent from the patients for research use was obtained prior to the investigation. Human PDAC models were collected during the first funding period of CRU5002, from July 2020 to June 2024, at the University Medical Center Göttingen (UMG).

### 2.2 Compounds

The cherry‐picked drug library (stock 10 mm, dissolved in DMSO), Sotorasib (AMG‐510) (#S8830), Batoprotafib (TNO155) (#S8987), Decitabine (#S1200), Ceralasertib (AZD8738) (#S7693), BI‐3406 (#S8916), MS023 (#S8112), and Nintedanib (#S1010) were purchased from Selleckchem (Cologne, Germany). MTRX1133 was from MedChemExpress (Monmouth Junction, NJ, USA). All compounds were solubilized in DMSO.

### 2.3 Statistics

All experiments were conducted in biological triplicates unless otherwise stated. In all figures, the mean and the standard deviation (SD) are depicted. Analysis of Variance (ANOVA) with correction for multiple testing was used to calculate *P*‐values. Analysis was performed with graphpad prism5/8/9 (RRID: SCR_002798; GraphPad Software, Boston, CA, USA). Tests were performed on non‐normalized data.

### 2.4 Cell lines

The human pancreatic cell line MiaPaCa2 (CRM‐CRL‐1420, RRID: CVCL_0428) and PSN1 (CRM‐CRL‐3211, RRID: CVCL_4892) cells were cultured in Dulbecco's Modified Eagle's Medium–high glucose (#D5796; Sigma‐Aldrich, Taufkirchen, Deutschland), AsPC1 (RRID: CVCL_0152) cells in RPMI‐1640 medium (#R8758; Sigma‐Aldrich), both supplemented with 10% (v/v) fetal calf serum (FCS) (#TMS‐013‐B; Merck Millipore, Berlin, Germany) at 37 °C in 5% CO_2_. The cell lines were sourced from the Clinic for Internal Medicine II at the Technical University of Munich and transferred to University Medical Center Göttingen 2021 (AsPC1, MiaPaCa2, PSN1). The human patient‐derived cell line (PDCL) 51T‐2D was generated from a patient‐derived organoid (PDO)‐51T and cultured in RPMI‐1640 Medium (#R8758; Sigma‐Aldrich) supplemented with 10% (v/v) FCS (#TMS‐013‐B; Merck Millipore). All experiments in the PDCL line were conducted using passages between 10 and 30. Murine PDAC cell lines were established from autochthonous Kras^G12D^‐driven cancer as described [7, 8, 9]. The murine cell lines 8661 and 8248 were cultivated in high glucose DMEM medium (#D5796; Sigma‐Aldrich) with 10% (v/v) fetal calf serum (FCS, #TMS‐013‐B; Merck Millipore).

### 2.5 Cell lines, mycoplasma test, and authentication

Human PDAC cell lines were authenticated by Single Nucleotide Polymorphism (SNP)‐Profiling conducted by Multiplexion in 2023 (Multiplexion GmbH, Heidelberg, Germany). PDAC cell lines were screened for *Mycoplasma* contamination using a recently described PCR‐based protocol [10] with the following primers: 5′ Primer 1: 5′CGCCTGAGTAGTACGTTCGC3′; 5′ Primer 2: 5′CGCCTGAGTAGTACGTACGC3′; 5′ Primer 3: 5′TGCCTGGGTAGTACATTCGC3′; 5′ Primer 4: 5′TGCCTGAGTAGTACATTCGC3′; 5′ Primer 5: 5′CGCCTGAGTAGTATGCTCGC3′; 5′ Primer 6: 5′CACCTGAGTAGTATGCTCGC3′; 5′ Primer 7: 5′CGCCTGGGTAGTACATTCGC3′; 3′ Primer 1: 5′GCGGTGTGTACAAGACCCGA3′; 3′ Primer 2: 5′GCGGTGTGTACAAAACCCGA3′; 3′ Primer 3: 5′GCGGTGTGTACAAACCCCGA3′. All experiments were performed in mycoplasma‐free cells.

### 2.6 KRAS sequencing

Sanger sequencing was used to confirm the *KRAS* ^*G12C*^ mutation of PDO‐51T and 51T‐2D‐PDCL. DNA was isolated using QIAamp® DNA Mini Kit (#51306; QIAGEN, Hilden, Germany). KRAS exon 2 was amplified by PCR using Phusion® High‐Fidelity DNA Polymerase (#M0531S; New England Biolabs, Frankfurt am Main, Germany) with the following primers: hKRAS_ex2_flank_Fw: 5′GGTACTGGTGGAGTAT TTGATAGTG 3′ and hKRAS_ex2_flank_Rv 5′ GGTCCTGCACCAGTAATATGCA 3′. PCR products were sequenced by Microsynth AG (Göttingen, Germany), and results were aligned to the wild‐type *KRAS* gene sequence (NG_007524.2) using the software snapgene viewer (version 6.0.2, Boston, MA, USA).

### 2.7 Combinatorial drug screen

MiaPaCa2 cells were seeded at a density of 1000 cells/well in 96‐well plates (#136101; Thermo Scientific™, Waltham, MA, USA) with 100 μL Dulbecco's Modified Eagle's Medium‐high glucose (#D5796; Sigma‐Aldrich) supplemented with 10% (v/v) fetal calf serum (FCS) (#TMS‐013‐B; Merck Millipore). After 24 h, cells were treated with a compound library consisting of 126 drugs. A liquid handling manual pin tool (#AFIX96FP; V&P Scientific, San Diego, CA, USA) was used to add each drug either as a single compound in a 7‐point dilution (3‐fold dilution series, highest concentration 10 μm) or in combination with Sotorasib (fixed anchored concentration of 6 nm). Following 72 h of treatment, cell viability was assessed by adding 25 μL of CellTiter‐Glo® Luminescent Assay (#G7573; Promega, Walldorf, Germany). Samples were incubated for 30 min on an orbital shaker, and luminescence was measured using a VICTOR™ X4 2030‐0040 Multilabel Plate Reader (PerkinElmer Cellular Technologies Germany GmbH, Hamburg, Germany). Dose–response curves were analyzed regarding the half maximal growth inhibitory concentration (GI_50_) and area under the curve (AUC) using the r package grmetrics [11]. Hits were defined by an ΔAUC < −0.1 and/or log_10_FC of the IC_50_ < −0.3. In addition, hits with an inappropriate curve fitting were excluded. For the MRTX1133‐anchored screening KRAS^G12D^‐mutated AsPC‐1 cells were seeded at a density of 1500 cells/well in white 96‐well plates (#136101; Thermo Scientific™) in 100 μL of RPMI‐1640 medium (#R8758; Sigma‐Aldrich) containing 10% (v/v) of fetal calf serum (FCS) (#TMS‐013‐B; Merck Millipore). After 24 h of cultivation, cells were treated with a compound library consistent of *n* = 139 drugs as described for the Sotorasib‐anchored screen. MRTX1133 was used in a fixed anchor concentration of 35 nm. End point measurement and analytics were the same as described for the Sotorasib‐anchored screen.

### 2.8 Clonogenic assay

MiaPaCa2 and 51T‐2D‐PDCL cells were seeded in 24‐well plates at a density of 3000 cells/well or 15 000 cells/well, respectively. After 24 h, cells were treated with drugs or combinations of drugs. After 7 days, the medium was removed, and wells were washed twice with 1× PBS. Cells were stained with 0.2% Crystal Violet solution (#T123.3; Carl Roth GmbH + Co. KG, Karlsruhe, Germany) for 15 min on an orbital shaker at room temperature. Following this, cells were washed four times with dH_2_O and allowed to dry. After 24 h, Crystal Violet dye was solubilized in 1% SDS solution (#CN30.3; Carl Roth GmbH + Co. KG), and the absorbance was measured at 570 nm using a VICTOR™ X4 2030‐0040 Multilabel Plate Reader. Quantified values were used to calculate a Bliss score with the synergy finder platform 2.0 (https://synergyfinder.fimm.fi/) [12].

### 2.9 Patient‐derived organoids (PDOs): generation and cultivation

The primary patient‐derived PDAC organoids (PDO‐51T, PDO‐16T) were isolated from resected primary pancreatic cancer following an established protocol [13] and in the framework of the Clinical Research Unit (CRU) 5002. In brief, the excised tumor was transferred to a tissue culture dish and minced into small fragments using a scalpel. The small tissues were transferred into a 15 mL conical tube containing Human Digestion Medium [Human Complete Feeding Medium (Advanced DMEM/F‐12 medium (#12634028; Gibco, Schwerte, Germany), containing 10 nm HEPES (#15630080; Gibco), 1×‐GlutaMAX (#35050061; Gibco), 0.1% BSA (#A9576; Sigma‐Aldrich), 10% R‐spondin1‐Conditioned medium (R‐spondin1—expressing HEK293T‐HA‐RspoI‐FC), 50% v/v Wnt‐3a‐Conditioned medium (Wnt3a‐expressing cells—HEK293‐WNT3A‐AFAMIN cells [14]), 1×‐B27 (#17504001; Thermo‐Fischer), 10 nm Nicotinamide (#N0636; Sigma‐Aldrich), 1.25 mm *N*‐acetylcysteine (#A9165; Sigma‐Aldrich), 100 μg·mL^−1^ Primocin (#ant‐pm‐2; Invivogen, Toulouse, France), 100 ng·mL^−1^ mNoggin (#250‐38; Peprotech, Hamburg, Germany), 50 ng·mL^−1^ hEGF (#PHG0313; Invitrogen), 100 ng·mL^−1^ hFGF10 (#100‐26; Peprotech), 10 nm hGastrinI (#3006; Tocris, Wiesbaden‐Nordenstadt, Germany), 500 nm A83‐01 (#2939; Tocris) supplemented with 5 mg·mL^−1^ Collagenase Crude Type XI (#C9407; Sigma‐Aldrich) and 10 μg·mL^−1^ DNase I (#D5025; Sigma‐Aldrich) and 10.5 μm Y‐27632 (#Y0503; Sigma‐Aldrich)]. Samples were subjected to 15‐min digestion at 37 °C in a rotator set to 300 r.p.m. After incubation, the larger tissue chunks were allowed to settle, and the supernatant (first fraction) was transferred into a new 15 mL conical tube. Fresh Human Digestion Medium was added to the original tube containing small undigested tissue fragments. The digestion process was repeated, and the supernatant (first fraction) was combined with the second fraction. The resultant combined fractions underwent centrifugation at 500 ***g*** for 5 min at 4 °C. If necessary, samples were incubated with ACK lysis buffer (#A10492‐01; Gibco) at room temperature for at least 3 min at room temperature to facilitate the lysis of red blood cells. Samples were centrifugated at 500 ***g*** for 5 min at 4 °C, the supernatant was discarded, and the pellet was resuspended in Matrigel Growth Factor Reduced (GFR) Basement Membrane Matrix‐Phenol Red‐free—LDEV‐free (#356231; Corning, Taufkirchen, Deutschland). Fifty microliter Matrigel domes were plated into pre‐warmed 24 well plates. Plates were incubated in a tissue culture incubator at 37 °C for 30 min to allow Matrigel solidification. Five hundred microliter of warm Human Complete Feeding Medium supplemented with 10.5 μm Y‐27632 was added after Matrigel solidification. Plates were incubated at 37 °C in 5% CO_2_.

For propagation, PDOs were cultured in cultivation media consisting of Advanced DMEM/F‐12 medium (#12634028; Gibco) supplemented with 10 mm HEPES (#15630080; Gibco), 1×‐GlutaMAX (#35050061; Gibco), and 0.1% BSA (#A9576; Sigma‐Aldrich), 10% R‐spondin1‐Conditioned medium (R‐spondin1‐Conditioned medium overexpressing cell line HEK293T), 1×‐B27 (#17504001; Thermo‐Fischer), 10 nm Nicotinamide (#N0636; Sigma‐Aldrich), 1.25 mm *N*‐acetylcysteine (#A9165; Sigma‐Aldrich), 100 μg·mL^−1^ Primocin (#ant‐pm‐2; Invivogen), 100 ng·mL^−1^ mNoggin (#250‐38; Peprotech), 100 ng·mL^−1^ hFGF10 (#100‐26; Peprotech), 10 nm hGastrin‐I (#3006; Tocris), and 500 nm A83‐01 (#2939; Tocris).

For passaging organoids, the matrix was mechanically disrupted by pipetting and collected in a 15 mL tube, which was kept on ice. The mixture was centrifugated for 5 min at 500 ***g*** at 4 °C. The supernatant was removed, and the pellet was resuspended in Express Enzyme (#12605028; Gibco) supplemented with 10 μg·mL^−1^ DNAse I (#D5025; Sigma‐Aldrich) and 10.5 μm Rho Kinase Inhibitor (Y‐27632) (#Y0503; Sigma‐Aldrich). After 15 min at 37 °C the reaction was stopped by adding 5 mL of ice‐cold wash medium [Advanced DMEM/F‐12 medium (#12634028; Gibco), containing 10 mm HEPES (#15630080; Gibco), 1×‐GlutaMAX (#35050061; Gibco), 0.1% BSA (#A9576; Sigma‐Aldrich)]. Following this, samples were centrifuged at 500 ***g*** for 5 min at 4 °C. The supernatant was removed and the pellet containing dissociated organoids was resuspended in Cultrex Reduced Growth Factor Basement Membrane Extract, Type 2 (#3536‐005‐02; R&D Systems, Wiesbaden‐Nordenstadt, Deutschland). The organoid suspension was plated as 50 μL domes into pre‐warmed 24‐well plates. Plates were then incubated for 1 h at 37 °C for Cultrex solidification. Following, 500 μL of pre‐warmed Human Complete Feeding Medium (supplemented 10.5 μm Rho Kinase Inhibitor; Y‐27632) (#Y0503; Sigma‐Aldrich) was added to each well. Plates containing organoids were then cultivated at 37 °C in a 5% CO_2_.

### 2.10 Pharmacotyping of cell lines, PDOs, and live cell imaging

For determining viability and growth of MiaPaCa2 cells, 1000 cells were seeded in 100 μL medium in 96‐well white (#136101; Thermo Fisher) and 96‐well clear plates (#168055; Thermo Fisher). After 24 h of incubation at 37 °C in 5% CO_2_, cells were treated with 7‐point dilutions of Sotorasib. Cell viability was measured after 72 h of treatment using CellTiter‐Glo® Luminescent Assay (#G7573; Promega) using 25 μL per well of the reagent. For all cell viability assays, luminescence was measured using a VICTOR™ X4 2030‐0040 Multilabel Plate Reader (PerkinElmer Cellular Technologies Germany GmbH, Hamburg, Germany). For live cell imaging, clear plates were incubated for 7 days at 37 °C in 5% CO_2_ and imaged using Incucyte® SX5 Live‐Cell Analysis System (Sartorius, Göttingen, Germany). Confluency was analyzed with standard software (incucyte®live‐cell analysis Software, Göttingen, Germany) using default settings. Media and compounds were replaced every 3 days.

Patient‐derived organoids were digested to a single‐cell suspension using TrypLE Express Enzyme (#12605028; Gibco). For live cell imaging (Incucyte® SX5; Sartorius) in Fig. 2B–D, 384 well plates (#6236585; Greiner, Frickenhausen, Germany) were used. 1250 cells/well were mixed in a total volume of 50 μL/well containing 10% of Cultrex Reduced Growth Factor Basement Membrane Extract, Type 2 (#3536‐005‐02; R&D Systems) and seeded. After 24 h, cells were treated with a 7‐point dilution of Sotorasib or a combination of TNO155 and Sotorasib. Plates were incubated for 7 days without media exchange and imaged. incucyte standard software was used to determine confluency, which was transformed to relative growth.

For PDO viability assay corresponding to Fig. S1E, 1250 cells/well were embedded in a 10 μL dome Cultrex Reduced Growth Factor Basement Membrane Extract, Type 2 (#3536‐005‐02; R&D Systems) in the center of a 96‐well white plate. Organoids were incubated for 7 days at 37 °C in 5% CO_2_, and media changed 3 days after seeding. After 7 days of growth, PDOs were treated with 7‐point dilutions of Sotorasib for additional 72 h. Cell viability was assessed by adding 25 μL of CellTiter‐Glo® Luminescent Assay (#G7573; Promega) and luminescence was measured using a VICTOR™ X4 2030‐0040 Multilabel Plate Reader (PerkinElmer Cellular Technologies Germany GmbH).

### 2.11 Generation of 2D cell line from PDOs

To generate the 2D cell line 51T‐2D‐PDCL, PDO‐51T were treated with Cell Recovery Solution (#11543560; Corning Life Sciences, Amsterdam, Netherlands) for 10 min to separate the organoids from the Matrigel. After this step, ice‐cold 1×‐PBS was added, and the cell suspension was centrifugated at 500 r.p.m. at 4 °C for 5 min. The supernatant was removed, and the cell pellet was cultivated in RPMI GlutaMAX® (#61870036; Life Technologies, Darmstadt, Germany) supplemented with 20% (v/v) FCS (#TMS‐013‐B; Merck Millipore).

### 2.12 Viability determination of murine PDAC cell lines—cell titer blue assay

Murine epithelial cell line 8661 and mesenchymal cell line 8248 cells were seeded at a density of 1000 cells/well in 96‐well plates (#137101; Thermo Scientific™). After 24 h, cells were treated with TNO155, Nintedanib or BI3406 either as a single compound in a 7‐point dilution (3‐fold dilution series, highest concentration 10 μm) or in combination with MRTX1133 (2‐fold dilution series, highest concentration 23 nm). Following 72 h of treatment, cell viability was assessed by adding 10 μL of Resazurin Cell Viability Assay (#39905; SERVA, Heidelberg, Germany). Samples were incubated for 1 h at 37 °C, and fluorescence was measured using a VICTOR™ X4 2030‐0040 Multilabel Plate Reader. Quantified values were used to calculate a Bliss score with the synergy finder platform 2.0 (https://synergyfinder.fimm.fi/).

### 2.13 Cell cycle analysis

MiaPaCa2 were seeded in 6 cm dishes and incubated for 24 h. Afterward, cells were treated with Sotorasib. Twenty‐four hours after treatment adherent and floating cells were harvested. Cells were washed with 1× PBS and detached using 0.05% EDTA (#P10‐026100; PAN‐Biotech, Aidenbach, Germany). Floating and detached cells were combined and centrifuged at 161 x g 4 °C, for 5 min. The supernatant was removed, and the cell pellet was washed with 1×‐ice‐cold‐PBS. Subsequently, the cell pellet was fixed with 70% ice‐cold ethanol for 1 h on ice. After incubation, cells were washed twice with fluorescence‐activated cell sorting (FACS) buffer (1% FBS and 0.003% EDTA) and centrifuged. The cell pellet was resuspended in FACS buffer and treated with 1 mg·mL^−1^ of RNase A (DNase und protease‐free) (#EN0531; Thermo Fisher Scientific GmbH, Dreieich, Germany) for 1 h at 37 °C. After RNA digestion, cells were stained for 10 min with Propidium iodide (PI) solution (50 μg·mL^−1^) (#556463; Becton Dickinson, Heidelberg, Germany). FACS was done using CytoFLEX S V4‐B2‐Y0‐R3 Flow Cytometer (Beckman Coulter GmbH, Krefeld, Germany). Data analysis was performed using flowjo 10 and graphpad prism 9 (Ashland, OR, USA).

### 2.14 Caspase 3/7 assay

1000 MiaPaCa2 cells and 3000 51T‐2D cells were seeded in 100 μL of growth medium in a white 96‐well plate. Following 24 h of incubation, cells were treated with vehicle (DMSO), Sotorasib (24 nm), and TNO155 (370 nm), either as a single compound or in combination. Caspase‐Glo® 3/7 3D assay (#G8982; Promega) was performed according to the manufacturer's instructions after 24 and 72 of treatment. Each experiment was conducted as three technical replicates and three biological replicates. Data analysis was conducted using graphpad prism 9.

### 2.15 Annexin V/PI staining

MiaPaCa2 and 51T‐2D cells were seeded in 6 cm dishes and allowed to adhere for 24 h. Subsequently, cells were treated with vehicle (DMSO), Sotorasib (24 nm), and TNO155 (370 nm) as single compounds and in combination. Adherent and floating cells were collected after 24 and 72 h after treatment. Cells were rinsed with 1× PBS and detached using 0.25% Trypsin–EDTA (#25200‐072; Gibco). Trypsinization was stopped by adding culture media containing 10% FBS (#F7524; Sigma‐Aldrich). Floating and detached cells were combined and centrifuged at 1000 r.p.m., 4 °C, for 5 min. The supernatant was aspirated, and the cell pellet was washed twice with ice‐cold 1×‐PBS. Cell pellets were then stained with a FITC‐conjugated Annexin V Apoptosis Detection Kit I (#556547; BD Pharmingen™, Heidelberg, Germany), according to the manufacturer's instructions. Apoptotic cell death was assessed using CytoFLEX S V4‐B2‐Y0‐R3 Flow Cytometer (Beckman Coulter GmbH). Early apoptotic cells were identified as Annexin V‐positive, whereas late apoptotic cells were defined as positive for both Annexin V and PI positive. Data analysis was conducted using flowjo 10 and graphpad prism 9.

### 2.16 Immunohistochemistry of PDO

51T‐PDO were seeded in 24 well plates and grown for 7 days. Subsequently, organoids were treated with vehicle (DMSO), Sotorasib (24 nm), and TNO155 (124 nm) as single compounds and in combination. After 24 and 72 h of treatment, organoids were collected in a Cell Recovery Solution (#11543560; Corning Life Sciences) to remove the Cultrex Reduced Growth Factor Basement Membrane Extract, Type 2 (#3536‐005‐02; R&D Systems). Organoids were collected and centrifuged at 500 r.p.m., 4 °C, for 5 min. Organoid pellets were fixed overnight in 4% formaldehyde (PanReac, AppliChem GmbH, Darmstadt, Germany). Subsequently, organoids were first embedded in HistoGel (Thermo Scientific), followed by dehydration, paraffin embedding, and sectioning (2 μm thickness, Thermo Scientific Microm HM430).

Paraffin‐embedded organoid sections were deparaffinized in xylol (#8118; J.T. Backer, Schwerte, Germany), followed by a descending series of ethanol (from 100% to 75% ethanol). Then, slides were incubated for 1 min in eosin (#10177; Süsse, Gudensberg, Germany) and dehydrated in increasing ethanol series (from 75% to 100% ethanol) and xylol (#8118; J.T. Backer). Slides were heated at 100 °C in a steamer (Braun, Melsungen, Germany) in Tris–EDTA (pH 8.5) for 20 min. Endogenous peroxidase activity was blocked using 3% hydrogen peroxide (in deionized water) for 15 min. Non‐specific antibody binding was blocked with 5% BSA in PBS for 15 min and subsequently washed with tris‐buffered saline containing 0.1% TritonX100 (TBST). Sections were incubated with the primary antibodies Ki67 (Invitrogen Cat# MA5‐14520, RRID: AB_10979488; 1 : 500) and Cleaved Caspase‐3 (Asp175) (5A1E) (Cell Signaling Technology; Cat# 9664 (also 9664P), RRID: AB_2070042, 1 : 200). Slides were incubated 10 min with Bright‐DAB (#BS04‐110A; ImmunoLogic, NL 1 : 25) and rinsed twice in deionized water. Subsequently, slides were immersed in Meyer's hematoxylin (#10231; Süsse) and washed for 10 min with tap water. Finally, samples were dehydrated by incubation in increasing ethanol series (from 75% to 100% ethanol) and xylol (#8118; J.T. Backer). After staining, slides were mounted using Vitro‐Clud® (Langenbrinck, Emmendingen, Germany). All samples were digitalized using Olympus IX83 (Olympus Life Science, Hamburg, Germany) using 100× magnification.

### 2.17 Protein extraction and western blotting

Cells were seeded into 10 cm dishes and treated with compounds for the indicated time points. After cell treatment, the cells were washed twice with ice‐cold PBS. Whole cells lysates (WCL) were prepared using NP‐40 cell lysis buffer [1 m Tris–HCl, 5 m NaCl, 1% NP‐40 (#74385; Sigma‐Aldrich Chemie, Taufkirchen, Germany), pH 7.8], supplemented with 1 m Dithiothreitol (DTT) (#A2948; AppliChem), PhosphoSTOP (#4906837001; Roche Diagnostics, Mannheim, Germany), and Protease Inhibitor (#11836170001; Roche Diagnostics). Protein concentrations were determined using Roti®‐Quant reagent (#K015.1; Carl Roth GmbH + Co. KG). WCL were resuspended in 5×‐Lämmli buffer and heated to 95 °C for 5 min. Proteins were fractionated on SDS/PAGE and transferred onto a PVDF membrane (#200T.1; Carl Roth GmbH + Co. KG). Membranes were blocked in 5% milk in TBST (10 mm Tris–HCl pH 7.6, 150 nm NaCl, 0.05% Tween20) for 2 h. Afterward, the membranes were washed with TBS and incubated with primary antibodies: mouse anti‐KRAS (Cat# WH0003845M1, RRID: AB_1842235, dilution 1 : 1000; Sigma‐Aldrich Chemie GmbH), Rabbit monoclonal Phospho‐p44/42 MAPK (Erk1/2) (Thr202/Tyr204) (D13.14.4E) (Cat# 4370S, RRID: AB_2315112, dilution 1 : 2000; Cell Signaling), Rabbit monoclonal p44/42 MAPK (Erk 1/2) (137F5) (Cat# 4695S, RRID: AB_10693601, dilution 1 : 1000; Cell Signaling), mouse anti‐HSP90 alpha/beta (F‐8) (Cat# sc‐13119, RRID: AB_675659, dilution 1 : 1000; Santa Cruz, Dallas, Tx, USA) in 5% BSA (anti‐KRAS, p44/42 MAPK and phospho‐p44/42 MAPK) or milk (anti‐HSP90) in TBST at 4 °C overnight. First antibodies were detected by incubation with HRP‐conjugated secondary antibodies for 2 h, either goat anti‐Rabbit IgG (#R1364HRP, RRID: AB_10262463, dilution 1 : 10000, OriGene Technologies, Rockville, MD, USA) or rabbit anti‐mouse IgG (#R1253HRP, RRID: AB_10266129, dilution 1 : 10 000; OriGene Technologies). Signals were detected using Immobilon®Forte Western horseradish polymerase (HRP) substrate (Cat# WBLUF0500; Merck Millipore), and images were captured and quantified with the ChemiDoc™ MP Imaging System (Bio‐Rad, Feldkirchen, Deutschland).

### 2.18 RNA‐seq analysis and ssGSEA

RNASeq was performed at the NGS Integrative Genomics Core Unit, University Medical Center Göttingen (UMG). The raw reads are quality controlled using fastqc (RRID: SCR_014583), and checked for both microbial and mouse contamination using Kraken [15] (RRID: SCR_005484) and fastq screen [16] (RRID: SCR_000141) respectively. Next, the sequence reads are trimmed using trimmomatic [17] (RRID: SCR_011848), mapped to the reference genome (Ensembl, genome assembly GRCh38.p13) using star [18] (RRID: SCR_004463) and finally the features are quantified using htseq‐count [19] (RRID: SCR_011867). All results are gathered into a comprehensive report using multiqc [20] (RRID: SCR_014982).

For single sample Gene Set Enrichment Analysis (ssGSEA), the gsva package [21] was employed. Initially, gene attributes were retrieved from Ensembl using the biomart package [22, 23]. To ensure robustness and minimize noise, lowly expressed genes, where the sum of read counts across all samples was less than the number of samples in the dataset, were excluded. Next, library size normalization was employed using the calcNormFactors and cpm (method = “TMM”) functions from the edger package [24]. Furthermore, transcript length was considered by calculating reads per kilobase (RPK) for each gene, and then Transcripts Per Million (TPM) values were calculated, which were then employed in the gsva (method = “gsva”) function to evaluate pathway enrichment. HALLMARKs ssGSEA scores of *n* = 36 PDO lines were clustered using clustvis [25]: distance: Euclidean, clustering method: Ward.

### 2.19 Panel‐sequencing

Molecular characterization was conducted using a custom gene panel including 83 pancreatic cancer‐associated genes. Briefly, targeted multigene panel sequencing was performed on 200 ng genomic DNA. For library preparation, SureSelect™ XTHS target enrichment kit (Agilent) with enzymatic fragmentation was used following the manufacturer's protocol (Agilent, Santa Clara, CA, USA). Libraries were sequenced on an Illumina NextSeq 550 with 2.5 high output chemistry and 2 × 150 bp read length. sequence pilot (JSI Medical Systems GmbH, Ettenheim, Germany) Software was used to align sequences to a human reference sequence (hg19) and for variant calling. Variants were assessed according to the American College of Medical Genetics (ACMG) guidelines [26] to classify variants of uncertain significance (VUS), likely pathogenic or pathogenic variants.

## 3 Results

**Fig. 1:**
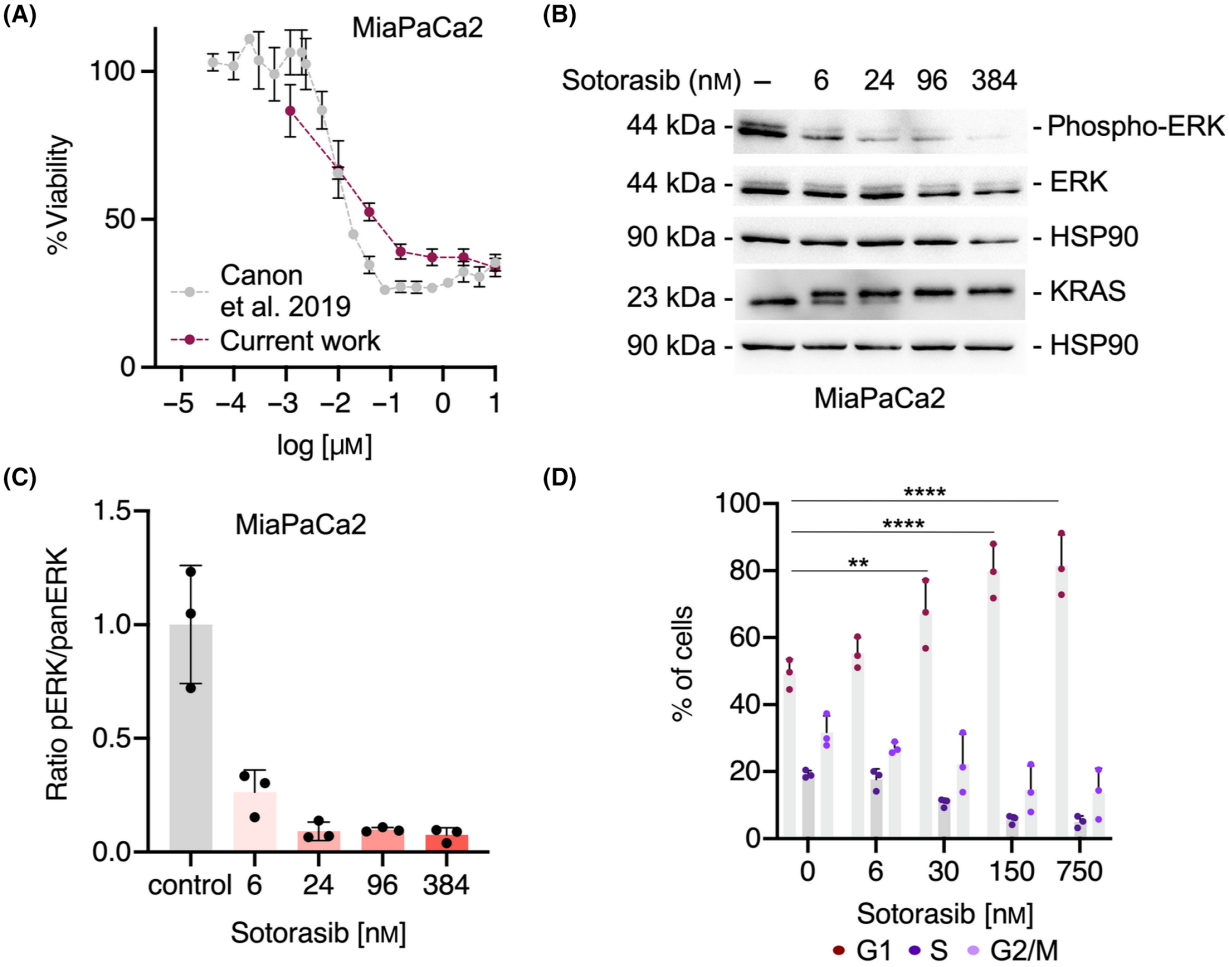
Sotorasib response of KRAS^G12C^‐mutated MiaPaCa2 cells. (A) MiaPaCa2 cells were treated with the indicated doses of Sotorasib and viability was determined 72 h after the treatment using ATP as a surrogate. *n* = 3. Gray line reproduced data from Canon et al. [27], Purple line: current manuscript. (B) Phospho‐ERK, ERK, and KRAS western blot 24 h after the treatment of MiaPaCa2 cell with the indicated doses of Sotorasib. HSP90: loading control. One representative blot out of three independent experiments (*n* = 3) is depicted. (C) Quantification of ERK phosphorylation corresponding to B. (D) Flow cytometry of propidium iodide (PI)‐stained cells 24 h after the treatment of MiaPaCa2 cells with the indicated doses of Sotorasib. *n* = 3. Two‐way ANOVA, Bonferroni correction: **P < 0.01, ****P < 0.0001. Mean and the standard deviation (SD) is shown.

To establish potential combination therapies based on KRAS^G12C^ inhibitors for PDAC, we used MiaPaCa2—a KRAS^G12C^ mutated line. Sotorasib is a clinical‐grade KRAS^G12C^ inhibitor that locks KRAS in an inactive GDP‐bound conformation. MiaPaCa2 cells responded to Sotorasib (Fig. 1A), but we observed residual growth after 7 days of treatment using live cell imaging (Fig. S1A). Compared to the 9 nm half‐maximal inhibitor concentration (IC_50_) reported by Canon et al. [27], we observed a similar dose–response curve with a calculated IC_50_ of 13 nm (Fig. 1A). To assess the on‐target activity of Sotorasib, we examined ERK phosphorylation 24 h after treatment. A distinct reduction in ERK phosphorylation was observed beginning at a concentration of 6 nm of Sotorasib (Fig. 1B,C). Furthermore, due to the covalent binding of Sotorasib to KRAS, we detected a mobility shift of the protein (Fig. 1B). We observed a cytostatic response characterized by the accumulation of cells in the G1‐phase of the cell cycle and a corresponding loss of the cells in the S‐phase and G2/M‐phase after 24 h of treatment (Fig. 1D).

**Fig. 2:**
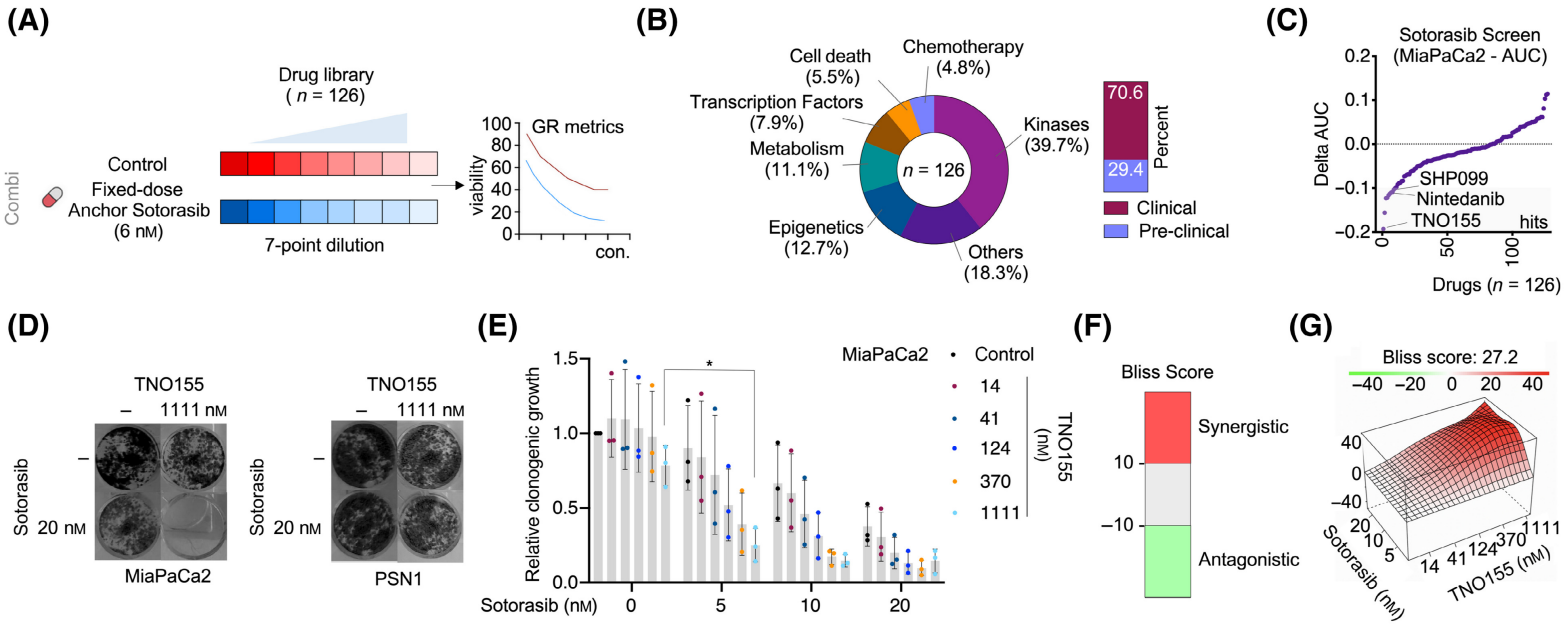
Drug Screen to find KRAS^G12C^ inhibitor combination. (A) Outline of the drug screening experiment. An anchored screen design (anchor: Sotorasib 6 nm) was used. Growth rate corrected analysis was done by the GR metrics algorithm. (B) Left panel: Illustration of the drug library target spectrum. Right panel: Seventy percent of the drugs are in the clinic (FDA‐approved in other tumor entities or at least in phase I trials). (C) Distribution of the delta area under the dose–response curve (AUC) values. A delta AUC < −0.1 was defined as one criterium for a hit. Three compounds with a delta AUC < −0.1 are depicted. (D) Clonogenic growth assay of MiaPaCa2 and PSN1 cells treated with the indicated doses and compounds. One representative clonogenic growth assay out of three is shown. (E) Quantification of clonogenic growth assays in MiaPaCa2 cells (*n* = 3). *One‐way ANOVA with Tukey's correction for multiple testing *P < 0.05. Mean and the standard deviation (SD) are depicted. (F) Illustration of Bliss synergy values: a score > 10 was considered synergistic, a score < −10 was considered antagonistic. (G) Bliss synergy score of the quantified clonogenic growth assays (E) of MiaPaCa2 cells was calculated with the synergy finder platform.

To identify potential combination therapies, we used a drug screen design, which was recently used to define MEK inhibitor (MEKi) combinations [28]. We applied an anchored drug screen with Sotorasib at a low constant dose of 6 nm (≈ IC_20_) (Fig. 2A). A compound library of *n* = 126 drugs covering a broad spectrum of targets was applied. 70% of the drugs are clinically approved for other cancers or at least in phase I testing (Fig. 2B). We treated the cells for 72 h and measured ATP as a surrogate of viability. Drug response curves were analyzed using the GR metrics pipeline [11].

**Fig. 3:**
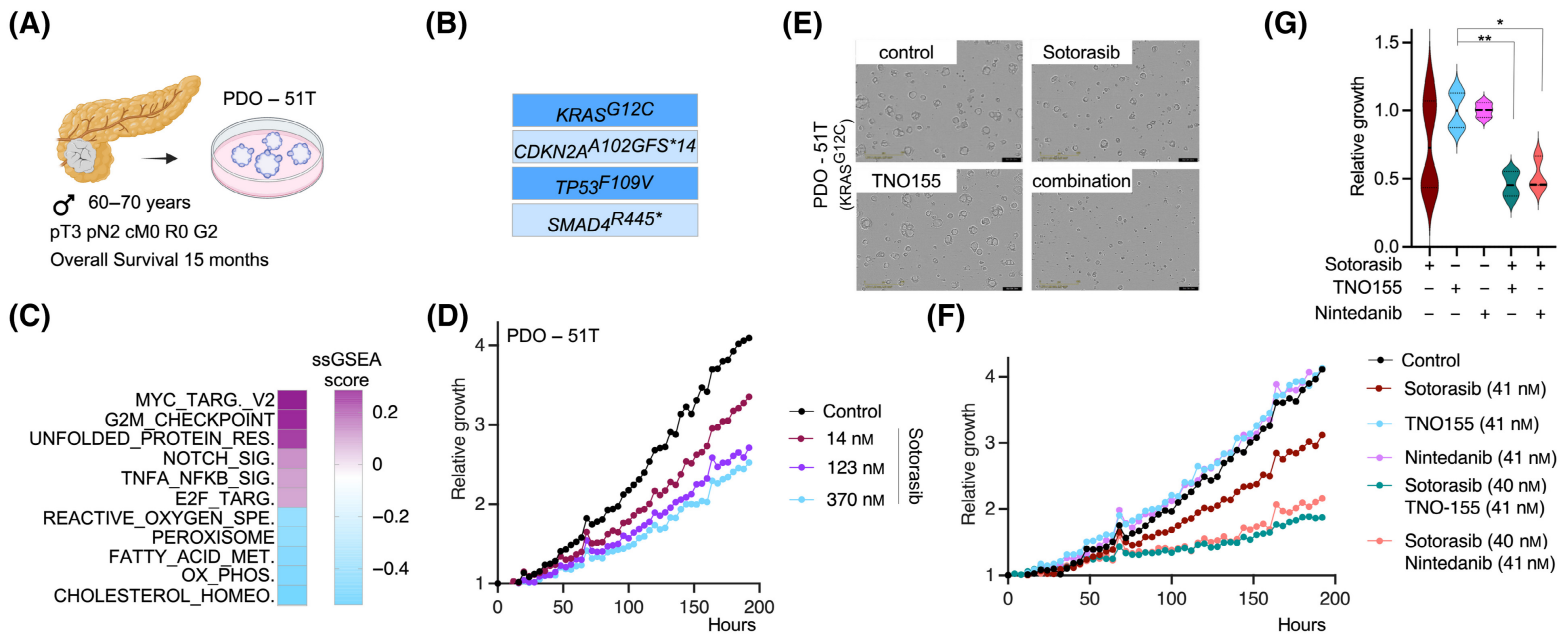
Efficacy of the TNO155 and Sotorasib combination in a *KRAS* ^*G12C*^‐mutated patient‐derived organoids (PDOs). (A) Case report of a *KRAS* ^*G12C*^‐mutated pancreatic ductal adenocarcinoma (PDAC) patient, from which the patient‐derived organoid (PDO) line PDO‐51T was isolated. After the resection of the tumor (pT3, pN2, cM0, R0, G2) the male patient was treated with adjuvant Gemcitabine/Capecitabine for 4 months. Afterwards, the patient was treated with mFOLFIRINOX and Gemcitabine/nab‐Paclitaxel. The overall survival was 15 months. (B) Results of the panel sequencing of PDO‐51T. (C) The top five enriched and depleted signatures of a single sample Gene Set Enrichment Analysis (ssGSEA) of the PDO‐51T line. (D) The indicated Sotorasib doses were used to determine growth over the indicated time points of PDO‐51T, measured by live cell imaging. *n* = 6. (E, F) PDO‐51T were treated with 41 nm Sotorasib (*n* = 6), 41 nm TNO155 (*n* = 6), 41 nm Nintedanib (*n* = 3), 40 nm Sotorasib/41 nm TNO155 (*n* = 6), and 40 nm Sotorasib/41 nm Nintedanib (*n* = 6) or left as vehicle controls (*n* = 6). Growth curves were determined by live cell imaging. Yellow scale bar: 400 μm. (G) Quantification of the growth after 192 h. Data of the experiment described in F were used to calculate the relative growth (ratio treatment groups versus control). One‐way ANOVA with correction for multiple testing according to Tukey: **P < 0.01, *P < 0.05. Median and quartiles are shown.

The delta of the area under the dose–response curve (AUC) distribution is depicted in Fig. 2C. To define hits, we employed the criteria of ΔAUC < −0.1 and/or log_10_FC of the IC_50_ < −0.3, in conjunction with the exclusion criteria for inappropriate curve fitting. Consequently, six hits were identified (Fig. S1B and Table S1): TNO155, an inhibitor of the tyrosine phosphatase SHP2 (SHP2i), encoded by the *PTPN11* gene; Nintedanib, a broad‐acting kinase inhibitor, targeting PDGFRB, FGFR1, DDR2 beyond others; Decitabine, a DNMT inhibitor; AZD6738, an ATR inhibitor; BI3406, a SOS1 inhibitor (SOS1i); and MS023, a type I protein arginine methyltransferases (PRMTs) inhibitor. We used long‐term clonogenic growth assays to validate the efficacy of the Sotorasib combinations (Fig. 2D and Fig. S1C,D). In this assay, we corroborated the value of the combination of Sotorasib with TNO155, BI3406, and Nintedanib. In PSN1 cells, harboring a *KRAS* ^*G12R*^ mutation, no synergism was observed, demonstrating specificity for *KRAS* ^*G12C*^ mutated cancers (Fig. 2D). Given that the allosteric SHP2i TNO155 emerged as the top hit in the screening experiment (Fig. 2C) and that SHP2i can be combined with inhibitors of the canonical KRAS signaling pathway with clinical activity [29], our focus centered on the TNO155‐Sotorasib combination. We quantified clonogenic growth assay data (Fig. 2E) and used the synergy finder platform to calculate a synergy score. We presumed a synergistic interaction if the Bliss score is larger than 10 (Fig. 2F) and observed a highly synergistic score (Bliss) of 27 in MiaPaCa2 cells (Fig. 2G). To further validate the combination, we aimed to analyze a patient‐derived organoid model (PDO). At least for chemotherapies, a concordance of responsiveness of PDOs *ex vivo* and in the clinic was reported [13, 30, 31]. As part of the Clinical Research Unit 5002 (CRU5002) an organoid biobank is currently established. In a male patient with a resectable PDAC (CRU5002 case: KFO‐TM047), the presence of a *KRAS* ^*G12C*^ driver mutation was observed and a PDO line was established and called PDO‐51T (Fig. 3A). The course of the disease is described in Fig. 3A and the post‐operative therapy in the corresponding figure legends. Considering the low frequency of *KRAS* ^*G12C*^ mutations, this PDO line is a rare and unique model. The PDAC harbors mutations in the classical PDAC genes *KRAS*, *TP53*, *CDKN2A*, and *SMAD4* (Fig. 3B) and we observed variants in other genes (Fig. S1E). Although documented in only a small number, the significant enrichment of mutation of the SWI/SNF chromatin remodeling complex member *ARID1A* in *KRAS* ^*G12C*^‐mutated PDACs was recently described [32]. Whereas *KRAS* ^*G12C*^ expressing MiaPaCa2 cells harbor an *ARID1A* mutation [33], no *ARID1A* mutation was detected in the PDO‐51T line. By utilizing single sample gene set enrichment analysis (ssGSEA) scores, we detected enrichment of pro‐proliferative and inflammatory signaling while observing depletion in depicted metabolic pathways in PDO‐51T (Fig. 3C). For comparison, the ssGSEA scores for a broader panel of PDAC PDOs (*n* = 36) are provided in Fig. S2. The *KRAS* ^*G12C*^ mutated PDO line showed a growth delay beginning with 14 nm of Sotorasib (Fig. 3D). In addition, we determined the dose–response curve of the PDO line using ATP as a readout for viability (Fig. S2B). In contrast, a *KRAS* ^*G12D*^ mutated PDO line showed no response to Sotorasib (Fig. S2B), demonstrating the specificity and pointing to a broad therapeutic window. We combined Sotorasib with TNO155 to assess the efficacy of the combination treatment using live cell imaging (Fig. 3E,F). Consistent with the data from MiaPaCa2 cells, we observed a significant growth delay with the combination (Fig. 3F,G). Additionally, we evaluated the combination of Sotorasib with Nintedanib in *KRAS* ^*G12C*^‐mutated organoids (Fig. 3F,G) and observed a comparable growth delay to that seen with the Sotorasib and TNO155 combination. Since the *KRAS* ^*G12C*^ allele is less common in PDAC, we aimed to determine if the combination is effective for other inhibitors. We evaluated the KRAS^G12D^ inhibitor MTRX1133 [34] in an unbiased drug screening experiment using the KRAS^G12D^ mutated PDAC cell line AsPC1 (Fig. S3A,B). Here, EGFR family inhibitors were prominent hits in the screen, but the primary hits from the Sotorasib screen, including TNO155, Nintedanib, and BI3406, also appeared in the MTRX1133 screen (Fig. S3A,B). Although we did not validate the hits in AsPC1 cells by clonogenic assays, we observed favorable synergy scores (Bliss) for MTRX1133 combinations with TNO155, Nintedanib, and BI3406 in murine PDAC cells (Fig. S3C–E). Therefore, we conclude that TNO155, Nintedanib, and BI3406 are relevant components in KRAS inhibitor‐based combinations, demonstrating efficacy across different species and inhibitors.

**Fig. 4:**
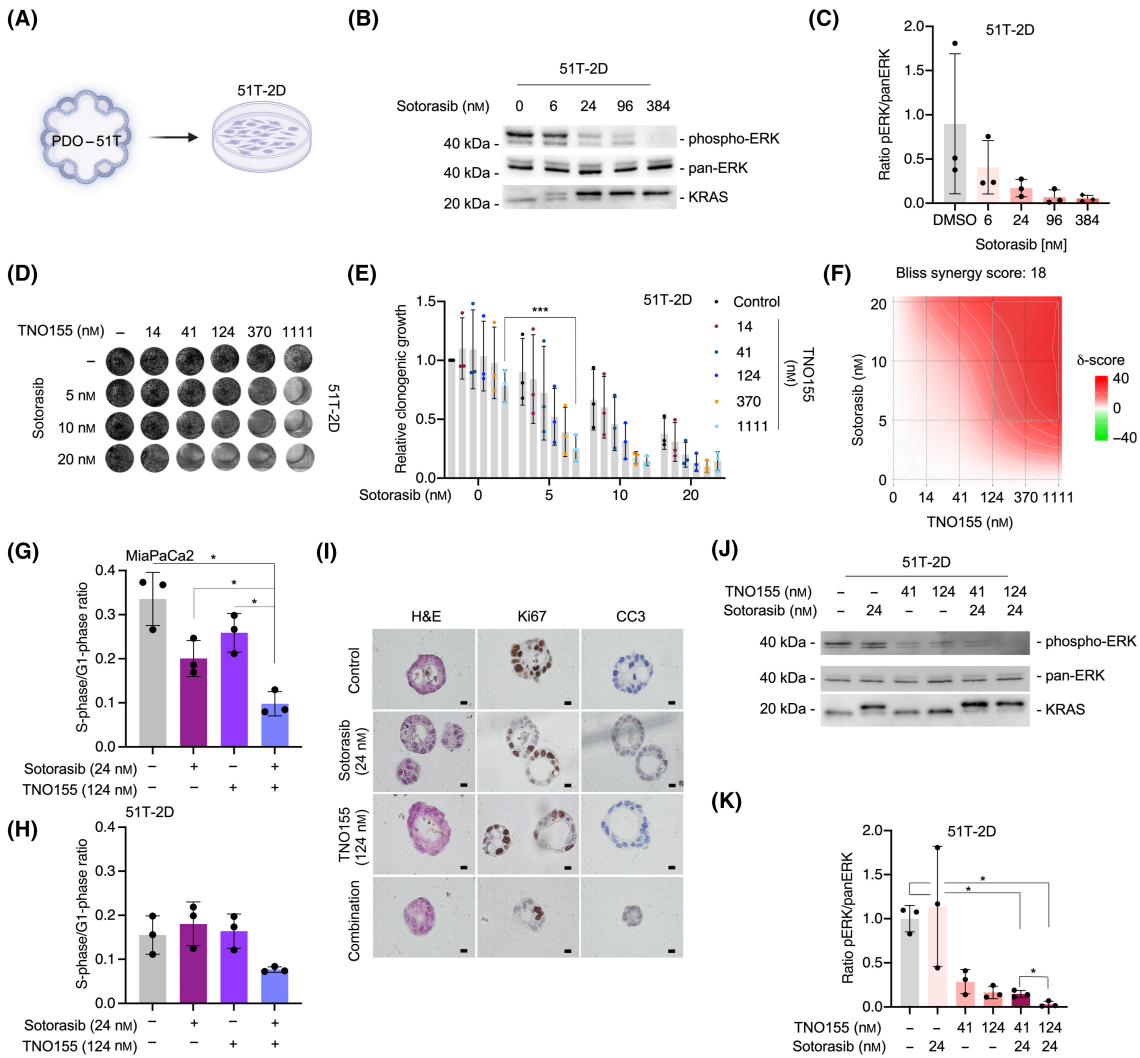
Synergism of Sotorasib with TNO155. (A) 2D pancreatic ductal adenocarcinoma (PDAC) cells (51T‐2D) were generated from PDO‐51T. (B) Phospho‐ERK, pan‐ERK, and KRAS western blot 24 h after the treatment of 51T‐2D cells with the indicated doses of Sotorasib. One representative blot out of three replicates (*n* = 3) is depicted. (C) Phospho‐ERK Quantification of three independent experiments (*n* = 3) corresponding to B. (D, E) Clonogenic growth assay of 51T‐2D cells treated with the indicated doses of TNO155 and Sotorasib. (D) One representative experiment. (E) Quantification of three independent experiments (*n* = 3). One‐way ANOVA with Tukey's correction for multiple testing: ***P < 0.001. (F) Bliss synergy score of the quantified clonogenic growth assays of 51T‐2D cells was calculated with the synergy finder platform. (G, H) MiaPaca2 and 51T‐2D cells were treated as indicated. After 72 h the cells were stained with propidium iodide (PI) and cell cycle FACS was performed. Depicted is the ratio of cells in the S‐phase of the cell cycle to cells in the G1‐phase (*n* = 3). One‐way ANOVA with correction for multiple testing according to Tukey: *P < 0.05. (I) Staining for Ki67 and cleaved caspase 3 (CC3) in PDO‐51T treated as indicated (*n* = 1). Scale bar = 10 μm. (J) Phospho‐ERK, pan‐ERK, and KRAS western blot 72 h after the treatment of 51T‐2D cells with the indicated doses of Sotorasib and/or TNO155. One representative blot out of three replicates (*n* = 3) is depicted. (K) Quantification of three independent experiments (*n* = 3) corresponding to J. One‐way ANOVA with Tukey's correction for multiple testing: *P < 0.05. Mean and the standard deviation (SD) are shown.

Additionally, we evaluated the effectiveness of the Sotorasib and TNO155 combination in a 2D PDAC line (51T‐2D), derived from the investigated *KRAS* ^*G12C*^ mutated PDO line (Fig. 4A). The on‐target activity was determined by investigating reduced phosphorylation of ERK (Fig. 4B,C) and the mobility shift of KRAS mediated by the covalent attachment of the inhibitor (Fig. 4B). Using 51T‐2D cells in clonogenic growth assays, we further confirmed the superiority of the TNO155 and Sotorasib combination therapy (Fig. 4D,E) and calculated a synergy score (Bliss) of 18 (Fig. 4F). To assess the cellular response induced by the combination therapy, we measured the activation of effector caspases 3 and 7. We observed a slight increase, although not statistically significant, in caspase 3/7 activity after 24 h of treatment with Sotorasib and TNO155 in MiaPaCa2 cells (Fig. S4A). However, no induction of caspase activity was detected in later treatment periods (Fig. S4A) or in 51T‐2D cells (Fig. S4B). Moreover, Annexin V FACS analysis did not reveal induction of apoptosis (Fig. S4C,D). In FACS analysis of propidium iodide‐stained cells, we observed an increased fraction of cells in the G1 phase of the cell cycle upon the combined treatment with Sotorasib and TNO155 (Fig. S4E,F), leading to a reduction in the S‐phase to G1‐phase ratio (Fig. 4G,H). Similarly, in the PDO‐51T line treated with the combination of Sotorasib and TNO155, no cleavage of caspase 3 was observed (Fig. 4I). While these data suggest an enhanced cytostatic response induced by the combination treatment with the analyzed concentration range, we cannot rule out the induction of apoptosis at different time points or doses, or the occurrence of other forms of cell death or fates. The potent inactivation of canonical KRAS signaling 72 h after the combination treatment mechanistically explains the observed synergism (Fig. 4J,K).

## 4 Discussion

Our screening experiment unbiasedly found SHP2i, SOS1i, and Nintedanib as combination partners for KRAS inhibitors in PDAC. The value of these inhibitors as partners for inhibitors of the canonical MEK–ERK pathway is evident. We recently described a Nintedanib‐MEKi combination with efficacy in *ex vivo* and *in vivo* PDAC models [28]. The approval of Nintedanib for forms of interstitial lung disease highlights its translational potential [35] and the kinase inhibitor is also being tested in cancers such as non‐small cell lung cancer (NSCLC) [36] and colorectal cancer (CRC) [37], among others. SHP2i acts synergistically with MEKi or ERKi in the context of PDAC [38, 39], leading to the implementation of clinical trials (e.g., SHERPA trial NCT04916236). Additionally, the combination of SHP2 inhibitors with KRAS^G12C^ inhibitors is currently being evaluated in clinical trials, as recently summarized [40]. These trials will yield valuable insights into both the efficacy and tolerability of this combination therapy. BI3406, which interferes with the interaction of SOS1 and KRAS, acts synergistically with MEKi *in vitro* and *in vivo* PDAC models [41]. BI3406 is also active in combination with a KRAS^G12C^ inhibitor in NSCLC and CRC models [42] and the combination regimen of a KRAS^G12C^ inhibitor with a SOS1i is under clinical testing (e.g., NCT04975256).

It is important to consider that the full efficacy of KRAS inhibitors *in vivo* depends on the immune system [43, 44]. Therefore, inhibiting canonical KRAS signaling, or using combinations based on these inhibitors, sensitizes tumor cells to immune checkpoint inhibitors, presenting an opportunity to explore such concepts in clinical settings [28, 44, 45, 46, 47].

KRAS^G12C^ inhibitors, as MEK inhibitors, induce rapid adaptive processes that ultimately limit their effectiveness [9, 48] and acquired resistance is characterized by mutations converging on the canonical KRAS pathway [49, 50, 51]. Adaptations involve various receptor tyrosine kinases (RTKs) and their ligands. In MiaPaCa2 cells treated with KRAS^G12C^ inhibitors, an upregulation of EGFR, FGFR3, IGFR1, MET, VEGFR1, and PDGFRA/B was observed [45], which leads to the restoration of canonical KRAS‐ERK signaling. Similar restoration of canonical KRAS signaling upon treatment with KRAS^G12C^ inhibitors by RTKs has been observed in other tumor types, such as colorectal cancer. This process involves the activation of wild‐type NRAS or HRAS [25]. Since SHP2 plays a central role in RTK signaling upstream of SOS1 and controls canonical KRAS signaling, our data might explain the molecular mechanisms underlying the synergy between our screening hits and Sotorasib. Consistently, the combination of Sotorasib and TNO155 led to a profound inactivation of canonical KRAS, as evidenced by reduced phosphorylation of ERK. Although the demonstration that reactivation depends on wild‐type RAS or KRAS^G12C^ in our models necessitates further experimentation, the potency of inhibitors targeting mutant and wild‐type inhibitors is evident. The RAS(ON) multi‐selective inhibitor RMC‐7977 interferes with active mutant and wild‐type GTP‐bound KRAS, HRAS, and NRAS [52]. RMC‐7977 has shown remarkable efficacy in preclinical PDAC models [53], while the clinical‐grade inhibitor RMC‐6236 induced a complete response in a stage IV PDAC patient [54]. Analysis of RMC‐7977 resistance revealed that alternative pathways, including copy number gains in MYC [53], can emerge if the RAS node is effectively blocked. This might suggest a need for alternative combination therapies with such inhibitors.

## 5 Conclusion

In summary, our study was driven by a drug screening experiment that revealed the potential of SOS1i, SHP2i, or broad‐spectrum kinase inhibitors, such as Nintedanib, as synergistic combination therapies with KRAS^G12C^ inhibitors in PDAC. Our findings could inform the design of future clinical trials and the work of molecular tumor boards.

## Declaration

Large language models (ChatGPT, Bard) and writing assistance (Grammarly) were used to improve readability and language. After using these tools, the author(s) reviewed and edited the content as needed and take(s) full responsibility for the content of the publication.

## Conflict of interest

The authors declare no conflict of interest.

## Author contributions

The work reported in the paper has been performed by the authors, unless clearly specified in the text. CTC, JDF, EH, and GSc contributed to conception and design of the study; CTC, JDF, D‐MS, CaS, LK, XF, DM, ED, ChS, SK, KM, JH, KC, GSa, NB, SR, MW, and GSc contributed to acquisition of data and/or analysis, curation, and interpretation of data; CTC, JG, BW, TB, MS, TDO, SK, GSa, US, MW, L‐CC, MR, VE, PS, MGh, MG, DS, EH, and GSc contributed to generation of important models and contribution of essential resources, technology, and funding; CTC, JDF, CaS, EH, and GSc contributed to drafting of the manuscript; all authors contributed to revision for important intellectual content and approval of the final version for publication.

## Supporting information


**Fig. S1.** Screening hits and validation.
**Fig. S2.** ssGSEA scores of the KFO5002 PDO biobank.
**Fig. S3.** MTRX1133‐anchored drug screen.
**Fig. S4.** Cell cycle arrest of KRASi‐SHP2i co‐treated pancreatic ductal adenocarcinoma (PDAC) cells.

**Table S1.** Drug screening results.

## Data Availability

The data generated in this study are available upon request from the corresponding author. Sequencing data are not publicly available due to privacy reasons of research participants.

## References

[1] Hofmann MH , Gerlach D , Misale S , Petronczki M , Kraut N . Expanding the reach of precision oncology by drugging all KRAS mutants. Cancer Discov. 2022;12(4):924–937. 35046095 10.1158/2159-8290.CD-21-1331 PMC9394389

[2] Drosten M , Barbacid M . Targeting KRAS mutant lung cancer: light at the end of the tunnel. Mol Oncol. 2022;16(5):1057–1071. 34951114 10.1002/1878-0261.13168 PMC8895444

[3] Bekaii‐Saab TS , Yaeger R , Spira AI , Pelster MS , Sabari JK , Hafez N , et al. Adagrasib in advanced solid tumors harboring a KRAS G12C mutation. J Clin Oncol. 2023;41(25):4097–4106. 37099736 10.1200/JCO.23.00434 PMC10852394

[4] Strickler JH , Satake H , George TJ , Yaeger R , Hollebecque A , Garrido‐Laguna I , et al. Sotorasib in KRAS p.G12C–mutated advanced pancreatic cancer. N Engl J Med. 2022;388(1):33–43. 36546651 10.1056/NEJMoa2208470 PMC10506456

[5] Sacher A , LoRusso P , Patel MR , Miller WH , Garralda E , Forster MD , et al. Single‐agent divarasib (GDC‐6036) in solid tumors with a KRAS G12C mutation. N Engl J Med. 2023;389(8):710–721. 37611121 10.1056/NEJMoa2303810

[6] Jin H , Wang L , Bernards R . Rational combinations of targeted cancer therapies: background, advances and challenges. Nat Rev Drug Discov. 2023;22(3):213–234. 36509911 10.1038/s41573-022-00615-z

[7] von Burstin J , Eser S , Paul MC , Seidler B , Brandl M , Messer M , et al. E‐cadherin regulates metastasis of pancreatic cancer in vivo and is suppressed by a SNAIL/HDAC1/HDAC2 repressor complex. Gastroenterology. 2009;137(1):361–371, 371.e1–5. 19362090 10.1053/j.gastro.2009.04.004

[8] Mueller S , Engleitner T , Maresch R , Zukowska M , Lange S , Kaltenbacher T , et al. Evolutionary routes and KRAS dosage define pancreatic cancer phenotypes. Nature. 2018;554(7690):62–68. 29364867 10.1038/nature25459 PMC6097607

[9] Schneeweis C , Diersch S , Hassan Z , Krauß L , Schneider C , Lucarelli D , et al. AP1/Fra1 confers resistance to MAPK cascade inhibition in pancreatic cancer. Cell Mol Life Sci. 2023;80(1):12. 10.1007/s00018-022-04638-y PMC9763154 36534167

[10] Orben F , Lankes K , Schneeweis C , Hassan Z , Jakubowsky H , Krauß L , et al. Epigenetic drug screening defines a PRMT5 inhibitor sensitive pancreatic cancer subtype. JCI Insight. 2022;7(10):e151353. 35439169 10.1172/jci.insight.151353 PMC9220834

[11] Clark NA , Hafner M , Kouril M , Williams EH , Muhlich JL , Pilarczyk M , et al. GRcalculator: an online tool for calculating and mining dose–response data. BMC Cancer. 2017;17(1):698. 29065900 10.1186/s12885-017-3689-3 PMC5655815

[12] Ianevski A , Giri AK , Aittokallio T . SynergyFinder 2.0: visual analytics of multi‐drug combination synergies. Nucleic Acids Res. 2020;48(W1):W488–W493. 32246720 10.1093/nar/gkaa216 PMC7319457

[13] Tiriac H , Belleau P , Engle DD , Plenker D , Deschênes A , Somerville T , et al. Organoid profiling identifies common responders to chemotherapy in pancreatic cancer. Cancer Discov. 2018;8(9):1112–1129. 29853643 10.1158/2159-8290.CD-18-0349 PMC6125219

[14] Mihara E , Hirai H , Yamamoto H , Tamura‐Kawakami K , Matano M , Kikuchi A , et al. Active and water‐soluble form of lipidated Wnt protein is maintained by a serum glycoprotein afamin/α‐albumin. Elife. 2016;5:e11621. 26902720 10.7554/eLife.11621 PMC4775226

[15] Wood DE , Salzberg SL . Kraken: ultrafast metagenomic sequence classification using exact alignments. Genome Biol. 2014;15(3):R46. 24580807 10.1186/gb-2014-15-3-r46 PMC4053813

[16] Wingett SW , Andrews S . FastQ screen: a tool for multi‐genome mapping and quality control. F1000Res. 2018;7:1338. 30254741 10.12688/f1000research.15931.1 PMC6124377

[17] Bolger AM , Lohse M , Usadel B . Trimmomatic: a flexible trimmer for Illumina sequence data. Bioinformatics. 2014;30(15):2114–2120. 24695404 10.1093/bioinformatics/btu170 PMC4103590

[18] Dobin A , Davis CA , Schlesinger F , Drenkow J , Zaleski C , Jha S , et al. STAR: ultrafast universal RNA‐seq aligner. Bioinformatics. 2013;29(1):15–21. 23104886 10.1093/bioinformatics/bts635 PMC3530905

[19] Anders S , Pyl PT , Huber W . HTSeq – a Python framework to work with high‐throughput sequencing data. Bioinformatics. 2014;31(2):166–169. 25260700 10.1093/bioinformatics/btu638 PMC4287950

[20] Ewels P , Magnusson M , Lundin S , Käller M . MultiQC: summarize analysis results for multiple tools and samples in a single report. Bioinformatics. 2016;32(19):3047–3048. 27312411 10.1093/bioinformatics/btw354 PMC5039924

[21] Hänzelmann S , Castelo R , Guinney J . GSVA: gene set variation analysis for microarray and RNA‐Seq data. BMC Bioinformatics. 2013;14(1):7. 23323831 10.1186/1471-2105-14-7 PMC3618321

[22] Durinck S , Moreau Y , Kasprzyk A , Davis S , Moor BD , Brazma A , et al. BioMart and Bioconductor: a powerful link between biological databases and microarray data analysis. Bioinformatics. 2005;21(16):3439–3440. 16082012 10.1093/bioinformatics/bti525

[23] Durinck S , Spellman PT , Birney E , Huber W . Mapping identifiers for the integration of genomic datasets with the R/Bioconductor package biomaRt. Nat Protoc. 2009;4(8):1184–1191. 19617889 10.1038/nprot.2009.97 PMC3159387

[24] Robinson MD , McCarthy DJ , Smyth GK . edgeR: a Bioconductor package for differential expression analysis of digital gene expression data. Bioinformatics. 2010;26(1):139–140. 19910308 10.1093/bioinformatics/btp616 PMC2796818

[25] Metsalu T , Vilo J . ClustVis: a web tool for visualizing clustering of multivariate data using principal component analysis and heatmap. Nucleic Acids Res. 2015;43(W1):W566–W570. 25969447 10.1093/nar/gkv468 PMC4489295

[26] Richards S , Aziz N , Bale S , Bick D , Das S , Gastier‐Foster J , et al. Standards and guidelines for the interpretation of sequence variants: a joint consensus recommendation of the American College of Medical Genetics and Genomics and the Association for Molecular Pathology. Genet Med. 2015;17(5):405–423. 25741868 10.1038/gim.2015.30 PMC4544753

[27] Canon J , Rex K , Saiki AY , Mohr C , Cooke K , Bagal D , et al. The clinical KRAS(G12C) inhibitor AMG 510 drives anti‐tumour immunity. Nature. 2019;575(7781):217–223. 31666701 10.1038/s41586-019-1694-1

[28] Falcomatà C , Bärthel S , Widholz SA , Schneeweis C , Montero JJ , Toska A , et al. Selective multi‐kinase inhibition sensitizes mesenchymal pancreatic cancer to immune checkpoint blockade by remodeling the tumor microenvironment. Nat Cancer. 2022;3(3):318–336. 35122074 10.1038/s43018-021-00326-1 PMC7612546

[29] Drilon A , Sharma MR , Johnson ML , Yap TA , Gadgeel S , Nepert D , et al. SHP2 inhibition sensitizes diverse oncogene‐addicted solid tumors to re‐treatment with targeted therapy. Cancer Discov. 2023;13(8):1789–1801. 37269335 10.1158/2159-8290.CD-23-0361 PMC10401072

[30] Hogenson TL , Xie H , Phillips WJ , Toruner MD , Li JJ , Horn IP , et al. Culture media composition influences patient‐derived organoids ability to predict therapeutic response in gastrointestinal cancers. JCI Insight. 2022;7(22):e158060. 36256477 10.1172/jci.insight.158060 PMC9746806

[31] Demyan L , Habowski AN , Plenker D , King DA , Standring OJ , Tsang C , et al. Pancreatic cancer patient‐derived organoids can predict response to neoadjuvant chemotherapy. Ann Surg. 2022;276(3):450–462. 35972511 10.1097/SLA.0000000000005558 PMC10202108

[32] Keane F , Chou JF , Walch H , Schoenfeld J , Singhal A , Cowzer D , et al. Precision medicine for pancreatic cancer: characterizing the clinico‐genomic landscape and outcomes of KRAS G12C‐mutated disease. J Natl Cancer Inst. 2024;djae095. 10.1093/jnci/djae095 PMC11378314 38702822

[33] Tomihara H , Carbone F , Perelli L , Huang JK , Soeung M , Rose JL , et al. Loss of ARID1A promotes epithelial–mesenchymal transition and sensitizes pancreatic tumors to proteotoxic stress. Cancer Res. 2021;81(2):332–343. 33158812 10.1158/0008-5472.CAN-19-3922 PMC8728103

[34] Wang X , Allen S , Blake JF , Bowcut V , Briere DM , Calinisan A , et al. Identification of MRTX1133, a noncovalent, potent, and selective KRASG12D inhibitor. J Med Chem. 2022;65(4):3123–3133. 34889605 10.1021/acs.jmedchem.1c01688

[35] Maher TM . Interstitial lung disease. JAMA. 2024;331(19):1655–1665. 38648021 10.1001/jama.2024.3669

[36] Yan S , Xue S , Wang T , Gao R , Zeng H , Wang Q , et al. Efficacy and safety of nintedanib in patients with non‐small cell lung cancer, and novel insights in radiation‐induced lung toxicity. Front Oncol. 2023;13:1086214. 37637045 10.3389/fonc.2023.1086214 PMC10449572

[37] Boland PM , Ebos JML , Attwood K , Mastri M , Fountzilas C , Iyer RV , et al. A phase I/II study of nintedanib and capecitabine for refractory metastatic colorectal cancer. JNCI Cancer Spectr. 2024;8(3):pkae017. 38697618 10.1093/jncics/pkae017 PMC11065487

[38] Frank KJ , Mulero‐Sánchez A , Berninger A , Ruiz‐Cañas L , Bosma A , Görgülü K , et al. Extensive preclinical validation of combined RMC‐4550 and LY3214996 supports clinical investigation for KRAS mutant pancreatic cancer. Cell Rep Med. 2022;3(11):100815. 36384095 10.1016/j.xcrm.2022.100815 PMC9729824

[39] Ruess DA , Heynen GJ , Ciecielski KJ , Ai J , Berninger A , Kabacaoglu D , et al. Mutant KRAS‐driven cancers depend on PTPN11/SHP2 phosphatase. Nat Med. 2018;24(7):954–960. 29808009 10.1038/s41591-018-0024-8

[40] Molina‐Arcas M , Downward J . Exploiting the therapeutic implications of KRAS inhibition on tumor immunity. Cancer Cell. 2024;42(3):338–357. 38471457 10.1016/j.ccell.2024.02.012

[41] Hofmann MH , Gmachl M , Ramharter J , Savarese F , Gerlach D , Marszalek JR , et al. BI‐3406, a potent and selective SOS1–KRAS interaction inhibitor, is effective in KRAS‐driven cancers through combined MEK inhibition. Cancer Discov. 2021;11(1):142–157. 32816843 10.1158/2159-8290.CD-20-0142 PMC7892644

[42] Thatikonda V , Lu H , Jurado S , Kostyrko K , Bristow CA , Bosch K , et al. Combined KRASG12C and SOS1 inhibition enhances and extends the anti‐tumor response in KRASG12C‐driven cancers by addressing intrinsic and acquired resistance. bioRxiv. 2023. 10.1101/2023.01.23.525210

[43] Kemp SB , Cheng N , Markosyan N , Sor R , Kim I‐K , Hallin J , et al. Efficacy of a small molecule inhibitor of KrasG12D in immunocompetent models of pancreatic cancer. Cancer Discov. 2022;13(2):298–311. 10.1158/2159-8290.CD-22-1066 PMC9900321 36472553

[44] Mahadevan KK , McAndrews KM , LeBleu VS , Yang S , Lyu H , Li B , et al. KRASG12D inhibition reprograms the microenvironment of early and advanced pancreatic cancer to promote FAS‐mediated killing by CD8+ T cells. Cancer Cell. 2023;41(9):1606–1620.e8. 37625401 10.1016/j.ccell.2023.07.002 PMC10785700

[45] Fedele C , Li S , Teng KW , Foster CJR , Peng D , Ran H , et al. SHP2 inhibition diminishes KRASG12C cycling and promotes tumor microenvironment remodeling. J Exp Med. 2020;218(1):e20201414. 10.1084/jem.20201414 PMC7549316 33045063

[46] Norgard RJ , Budhani P , O'Brien SA , Xia Y , Egan JN , Flynn B , et al. Reshaping the tumor microenvironment of KRASG12D pancreatic ductal adenocarcinoma with combined SOS1 and MEK inhibition for improved immunotherapy response. Cancer Res Commun. 2024;4(6):1548–1560. 38727236 10.1158/2767-9764.CRC-24-0172 PMC11191876

[47] Cortesi A , Gandolfi F , Arco F , Chiaro PD , Valli E , Polletti S , et al. Activation of endogenous retroviruses and induction of viral mimicry by MEK1/2 inhibition in pancreatic cancer. Sci Adv. 2024;10(13):eadk5386. 38536927 10.1126/sciadv.adk5386 PMC10971493

[48] Hallin J , Engstrom LD , Hargis L , Calinisan A , Aranda R , Briere DM , et al. The KRASG12C inhibitor MRTX849 provides insight toward therapeutic susceptibility of KRAS‐mutant cancers in mouse models and patients. Cancer Discov. 2020;10(1):54–71. 31658955 10.1158/2159-8290.CD-19-1167 PMC6954325

[49] Tanaka N , Lin JJ , Li C , Ryan MB , Zhang J , Kiedrowski LA , et al. Clinical acquired resistance to KRASG12C inhibition through a novel KRAS Switch‐II pocket mutation and polyclonal alterations converging on RAS–MAPK reactivation. Cancer Discov. 2021;11(8):1913–1922. 33824136 10.1158/2159-8290.CD-21-0365 PMC8338755

[50] Lietman CD , Johnson ML , McCormick F , Lindsay CR . More to the RAS story: KRAS G12C inhibition, resistance mechanisms, and moving beyond KRAS G12C. Am Soc Clin Oncol Educ Book. 2022;42(42):205–217. 10.1200/EDBK_351333 35561303

[51] Awad MM , Liu S , Rybkin II , Arbour KC , Dilly J , Zhu VW , et al. Acquired resistance to KRASG12C inhibition in cancer. N Engl J Med. 2021;384(25):2382–2393. 34161704 10.1056/NEJMoa2105281 PMC8864540

[52] Holderfield M , Lee BJ , Jiang J , Tomlinson A , Seamon KJ , Mira A , et al. Concurrent inhibition of oncogenic and wild‐type RAS‐GTP for cancer therapy. Nature. 2024;629(8013):919–926. 38589574 10.1038/s41586-024-07205-6 PMC11111408

[53] Wasko UN , Jiang J , Dalton TC , Curiel‐Garcia A , Edwards AC , Wang Y , et al. Tumor‐selective activity of RAS‐GTP inhibition in pancreatic cancer. Nature. 2024;629(8013):927–936. 38588697 10.1038/s41586-024-07379-z PMC11111406

[54] Jiang J , Jiang L , Maldonato BJ , Wang Y , Holderfield M , Aronchik I , et al. Translational and therapeutic evaluation of RAS‐GTP inhibition by RMC‐6236 in RAS‐driven cancers. Cancer Discov. 2024;14(6):994–1017. 38593348 10.1158/2159-8290.CD-24-0027 PMC11149917

