# Supplementary material for: KRASG 12C ‐inhibitor‐based combination therapies for pancreatic cancer: insights from drug screening
Source: Mol Oncol. 2024 Sep 10;19(2):295–310. doi: 10.1002/1878-0261.13725 (PMC11792994; doi:10.1002/1878-0261.13725)
Supplement: Supplementary file 1 — Fig. S1. Screening hits and validation. Fig. S2. ssGSEA scores of the KFO5002 PDO biobank. Fig. S3. MTRX1133‐anchored drug screen. Fig. S4. Cell cycle arrest of KRASi‐SHP2i co‐treated pancreatic ductal adenocarcinoma (PDAC) cells. [file MOL2-19-295-s002.pdf]

Supplemental Figure 1

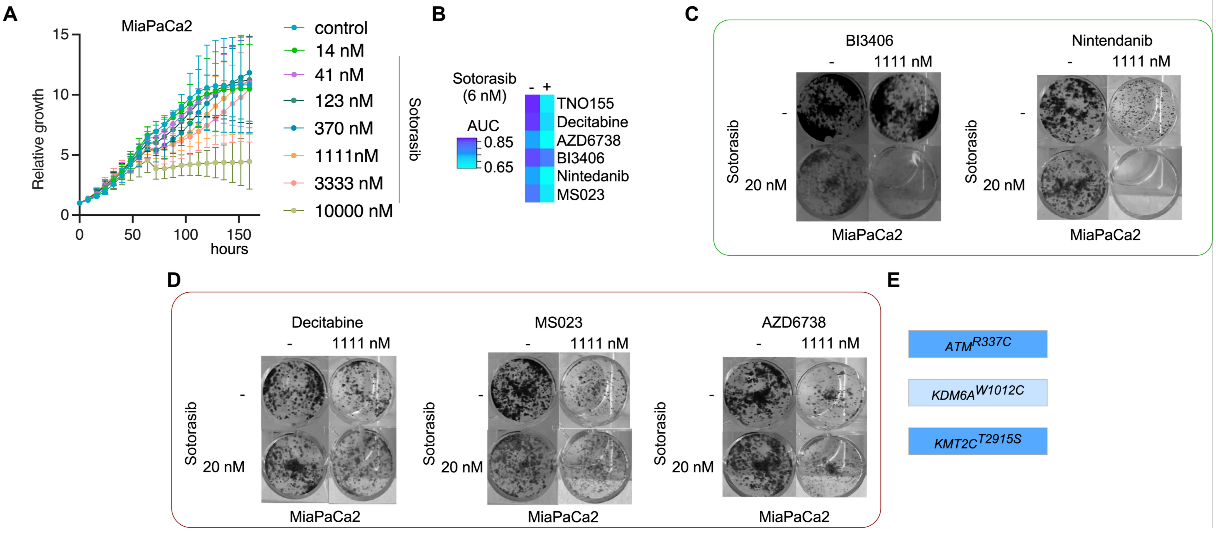

Supplemental Figure 1 Screening hits and validation.

A MiaPaCa2 cells were treated with the indicated doses of Sotorasib over time and monitored using live cell imaging. Medium with inhibitor was replenished after three days. Confluency was used as a surrogate of growth and used to calculate relative growth values. Experiment was done as three biological replicates (n = 3). **B** Screening hits of the Sotorasib anchored drug screen in MiaPaCa2 cells, defined by: delta AUC<-0.1 and/or log10FC<-0.3 and manual exclusion of hits with inappropriate curve fitting. The AUC values were color coded. **C** and **D** Clonogenic growth assay of MiaPaCa2 cells treated with the indicated doses and compounds. Green box: successful validation, Red box: failed validation. n = 3. **E** Additional variants of uncertain significance (VUS) observed in PDO-51T.

Supplemental Figure 2

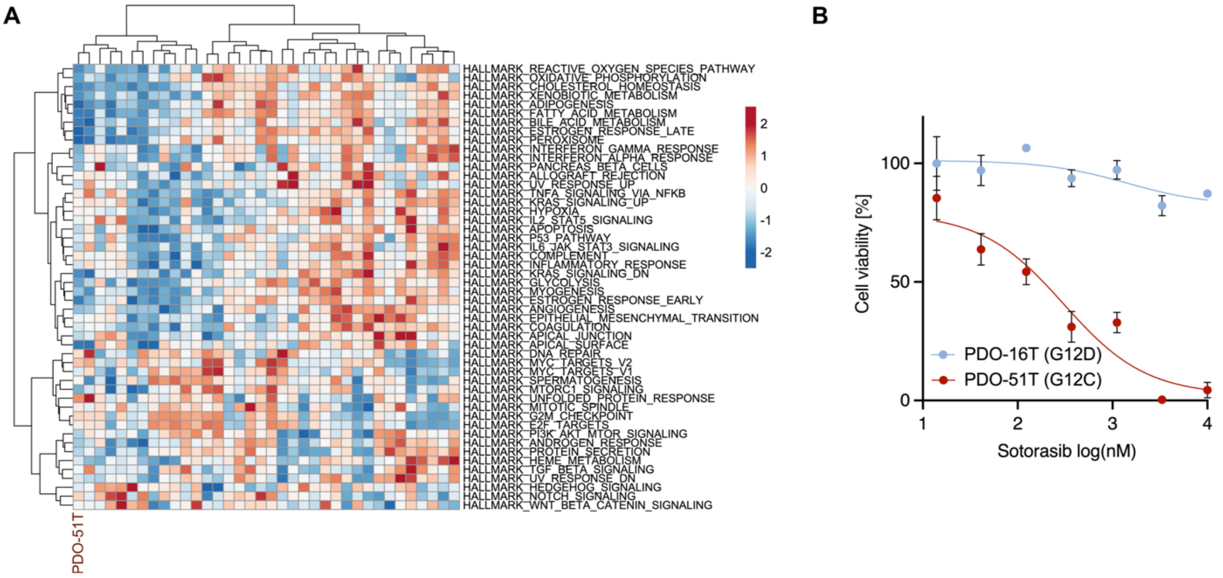

Supplemental Figure 2 ssGSEA scores of the KFO5002 PDO biobank.

A Single sample Gene Set Enrichment Analysis (ssGSEA) scores of the KFO5002 PDO biobank were clustered (distance: Euclidean, clustering method: Ward). The *KRAS*^{G12C}-mutated line (PDO-51T) is indicated. Legend: variance scaled ssGSEA score. **B** Sotorasib dose-response curves of PDO-16T with a *KRAS*^{G12D} and PDO-51T with a *KRAS*^{G12C} mutation. 10 µl domes were seeded in 96 wells. After 7 days of growth, established organoids were treated with the indicated doses of Sotorasib for 72 hours. Afterwards, viability was determined using ATP as a surrogated, which was determined by CellTiter-Glo® assay. n = 1, error bar from technical replication.

Supplemental Figure 3

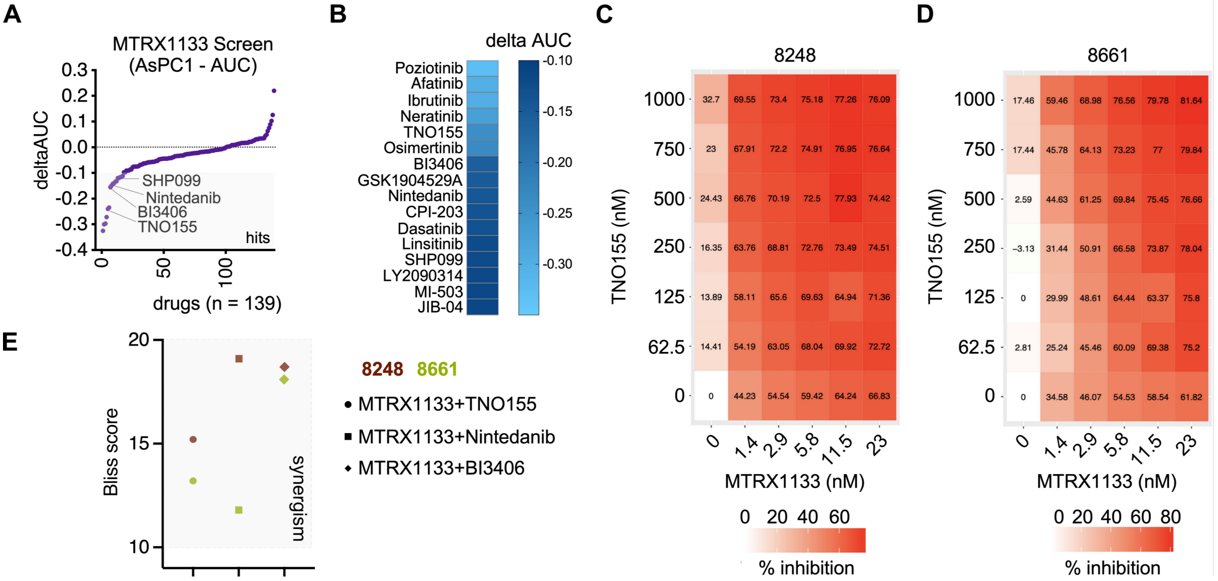

Supplemental Figure 3 MTRX1133-anchored drug screen.

A Distribution of the delta area under the dose-response curve (AUC) values in a drug screen in AsPC1 cells with MTRX1133 as an anchor (anchor concentration 35 nM). A delta AUC < -0.1 was defined as one criterium for a hit. SHP2i, SOS1i, and Nintedanib are depicted. **B** Heatmap of all screening hits with a delta AUC < -0.1 in AsPC1 cells. The delta AUC is color coded. **C** and **D** The murine PDAC cell line 8248 and 8661 were treated with the indicated treatments and concentrations for 72 hours. n = 3. Afterwards viability was determined and the percentage of inhibition is depicted and color coded. **E** The Bliss synergy score was calculated for the indicated cell lines and combinations. Values are based on combination treatments as indicated and exemplified in C and D. The area of synergism is depicted.

Supplemental Figure 4

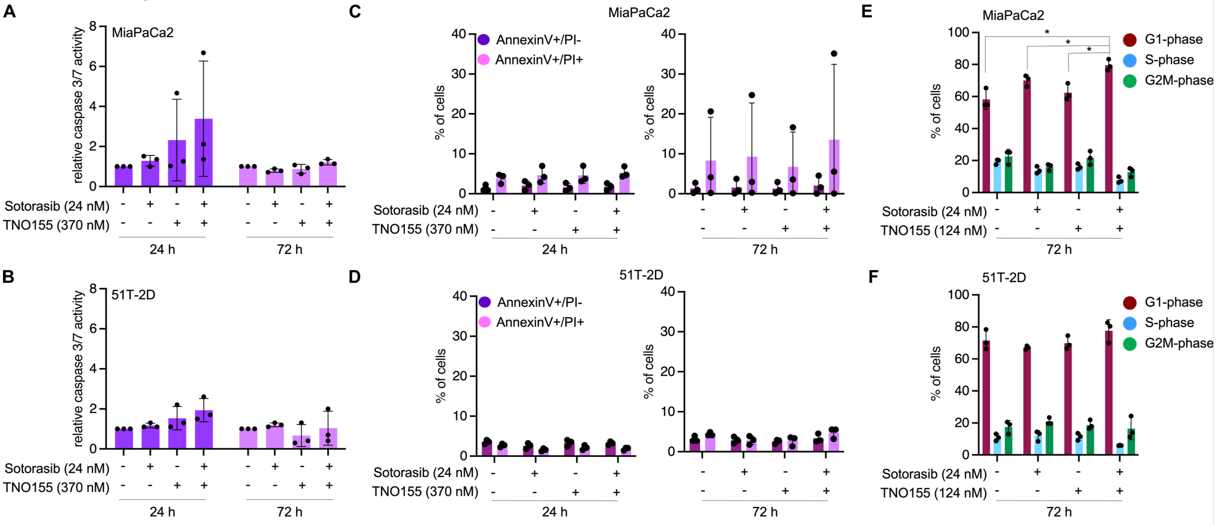

Supplemental Figure 4 Cell cycle arrest of KRASi-SHP2i co-treated pancreatic ductal adenocarcinoma (PDAC) cells.

A and **B** Caspase 3/7 activity of described human pancreatic ductal adenocarcinoma (PDAC) cells treated for 24 and 72 hours as indicated. n = 3. **C** and **D** Annexin V FACS analysis of described human PDAC cells treated for 24 and 72 hours with as indicated. Annexin V+/ propidium iodide (PI) -: early apoptosis, Annexin V+/PI+: late apoptosis. n = 3. **E** and **F** MiaPaca2 and 51T-2D cells were treated as indicated. After 72 hours the cells were stained with PI and cell cycle FACS was performed. (n = 3). Red: G1-phase, Blue: S-phase, Green: G2/M-phase. One-way ANOVA with correction for multiple testing according to Tukey: *p<0.05.
